# Stressful life events among adolescents: The development of a new measure

**DOI:** 10.4103/0019-5545.33255

**Published:** 2007

**Authors:** Shilpa Aggarwal, Col. H. R. A. Prabhu, Lt. Col. Aalok Anand, Lt. Col. Atul Kotwal

**Affiliations:** Department of Psychiatry, Base Hospital, Delhi Cantt, New Delhi - 110 010, India

**Keywords:** Adolescent, stressful life event, scale

## Abstract

**Background:**

Adolescence can be a stressful time for children, parents and adults who work with teens. We believe that a scale measuring the events perceived as stressful by an average Indian adolescent is necessary due to the presence of irrelevant items and absence of items related to our culture on foreign scales.

**Aim:**

This study was done to adapt and test the validity of a scale measuring stress caused due to life events in an Indian adolescent; to assess clinical value of the instrument in exploring causal relationships between stressful events and behavioral problems; and to compare the degree of overlap in stress-causing events between adolescents and their parents during the same timeframe.

**Materials and Methods:**

An adolescent life event stress scale (ALESS) containing 41 items was administered to 156 adolescents for formulation and 102 adolescents for validation. A third set of 112 adolescents was used to compare ALESS scores with child behavior checklist (CBCL) scores and parental stress scores due to life events.

**Results:**

The comparison showed a strong positive correlation with CBCL scores with a model fit (r^2^ = 0.32) and a weak positive correlation with parental stress (Pearson's coefficient = 0.011) due to life events.

**Conclusion:**

Thus, a life event scale for adolescents was especially adapted to the Indian conditions.

Adolescence can be a stressful time for children, parents and adults who work with teens. Children are dealing with the challenges of going through puberty, meeting changing expectations and coping with new feelings. Many also worry about moving from an elementary to a middle or junior high school. And some kids may have to deal with things that their peers don't have to face such as the death of a family member or moving to a new town. Most children meet these challenges successfully and grow into healthy adults while others have a harder time coping with their problems.

During adolescence, the onset of psychological disorders may be fast (days or weeks) or slow (months or years) depending in part on the nature of social adversities. What the exact negative psychological effects are and why the time of onset varies following exposure to negative circumstances, remain almost entirely unknown. An important assumption is that events and difficulties carry a latent and undesirable psychological construct (such as personal threat or negative impact to the self) that can be inferred from a detailed recall of the social characteristics of the experience. Recent advances in neurosciences have opened up possibilities for characterizing in a more direct way the intermediate mental and neural processes responsible for organizing behavioral responses to different forms of adversity.

Coping with social risks may depend on the presence of a sequential set of mental processes involving emotion recognition, appraisal of the implications for the self and initiation of control processes. These processes determine the form of a behavioral response, which could manifest as a behavioral problem and are measurable using various instruments like CBCL.[1] What constitutes a social adversity for an adolescent? An event which might be of utmost importance to an adult might be of no consequence to an adolescent. Even for adults, the distinctions among traumatic events, stressors and minor ‘hassles’ can be difficult to make.[2,3] It becomes more relevant in adolescents, thus making the measurement of the stress caused due to them, difficult.

Although there are foreign measures of potentially traumatic events, there is a dire necessity of a scale measuring the events perceived as stressful by an average Indian adolescent. There are many items on these foreign scales, which seem to be out of place in the Indian milieu, *e.g*., chronic car trouble. Also, since an average Indian adolescent remains emotionally and financially dependent on the parents, a need for inclusion of family / parent-related events was felt as they were found to be missing from western scales. This study was thus undertaken:

- To adapt and test the validity of a scale measuring stress caused due to life events in an Indian adolescent.
- To assess the clinical value of the instrument in exploring causal relationships between stressful events and behavioral problems.
- To compare the degree of overlap in stress-causing events between adolescents and their parents during the same timeframe.

The period of adolescence is one of rapid growth, change, relocation and self-discovery, which are defining qualities of stressful experience.[4] In theory, most prevailing models of developmental psychopathology recognize the potential importance of psychosocial stress in the etiology and maintenance of both internalizing and externalizing disorders in youth.[5–8] Both long-standing and recent social adversities precede and increase the risk for emotional and behavioral psychopathology during the school-age years.[9,10] The study of stressful life events and their impact upon the individual is fraught with numerous conceptual and methodological problems.[11–15]

Four important areas of concern have been identified: a) the role of personal control over event occurrence, b) the contamination between life changing events and the outcome variables (*e.g.* poor health, psychological distress), c) the need for consistent and reliable measures, and d) the necessity for cross-validating results. The specific nature of the change has been examined as a factor mediating the stressfulness of the event. For instance, the desirability[16] and the area of life[17,18] of the event occurrence have been considered important facets of whether the event is perceived and responded to, as stressful. On the other hand, controllability has been suggested as an important quality of an event with regard to the impact exerted upon the individual.[19]

If an event is under volitional control of the person, it should have less impact upon disequillibration as opposed to spontaneous or uncontrollable events which a person must inevitably face and bear. A great deal of criticism has been raised against stress research due to frequent confounding of the antecedent life events and subsequent stress reactions such as poor health, use of drugs, depression and psychosomaticism.[14,20] Thus, it is necessary to scrupulously determine that antecedent life events are not inadvertently included in or contaminated with the outcome measures. An increased incidence of psychiatric disorders in children exposed to markedly adverse circumstances of family life, including difficult socio-economic conditions, has led to the concept of the child at risk for psychiatric disorders.[21,22]

Vincent and Rosenstock's[23] study of inpatient adolescents showed that prior to hospitalization; those with psychiatric disorders had suffered more stressful events than those with physical disorders. On the other hand, Hudgens[24] noted a relationship between a group of personal stressors and depression in adolescents with medical disorders. Cohen Sandler *et al.*[25] retrospectively examined medical records to determine the amount of stress in suicidal, depressed and nondepressed children, finding significantly more stress in the suicidal group. A few studies have examined whether or not acute stressful events provoked or precipitated illness in children.

It was found that negative life events reported by parents were associated with children's psychological maladjustment and physical health problems.[26] Also, a significant relationship was found between major life events in the parents' lives and children's affective balance.[27] There was a positive correlation between stressful events reported by parents and depression in adolescents (especially boys) due to disruption in parenting practices.[28]

Processing affect-related, meaning-of-life events appears to be mediated by the medial prefrontal cortex functioning as the executive component for limbic-cortical activity. Although the basic programming of these neural networks is genetic, the fine-tuning most probably occurs through social experience in childhood and adolescence. Animal studies have shown deleterious consequences of social stress on neural structure and function, implicating an effect of the social environment on the brain through the physiological consequences of persistent interpersonal difficulties. Determining the relative effects of chronic and recent life events and difficulties on the patterning of psychological functions and their related neural structures is a major goal of future developmental research.

Such vertically integrated science will provide important clues about the interplay between social experiences, mental processes and their neural substrates. Instrumentation for measuring stress due to life events has been developed predominantly along the lines of respondent-based or checklist methods. One such respondent-based instrument, developed for use with children and adolescents, is the Life Event Record (LER) ([Coddington, 1972]).[29] The LER and variations of the LER, such as the Life Events Checklist (LEC) ([Johnson and McCutcheon, 1980]) have been widely used in studies of adverse events among children and adolescents with various psychiatric disorders. Validation of other life event scales, like the Tamil version of Impact of Event Scale[30] among adolescents have also been done in India.

The current study attempts to adapt the social readjustment rating scale (SRSS) to suit Indian conditions. Despite criticism, the SRRS is one of the most widely cited measurement instruments in stress literature.[31] Evaluation of content-related criticisms,including differential prediction of desirable compared to undesirable life events, controllable compared to uncontrollable life events and contaminated compared to uncontaminated life event items has been carried out in research conducted in recent years. Statistical data has proved the general consensus that the SRRS is a useful tool for stress researchers and practitioners.

## MATERIALS AND METHODS

Two different schools located in the Delhi Cantonment Area in New Delhi, were selected depending on the accessibility and their students. One school catered to the children of Officers in the Indian Army belonging to the upper middle class and upper class. The second school catered to the needs of Jawans in the Indian Army and civilians mostly belonging to the lower middle class and lower socioeconomic strata. A simple random sample of 170 students was then selected from standards 8^th^, 9^th^ and 11^th^. 10th and 12^th^ standard students were not included in the study due to academic pressure for these grades.

Permission to conduct the study was taken from the school authorities and informed consent was taken from the parents / guardians of these children. Guardians of 12% of the adolescents did not give informed consent and thus, these students were excluded from the study. The 41 item-containing adolescent life event stress scale based on Holmes and Rahe's[32] social readjustment rating scale and student stress scale[33] was administered to the students by the investigators using an “independent” measure. The measure was initially explained to the participants and the questionnaire was administered by giving a copy to each student to fill independently without any prompting from the investigators.

Students were to imagine that the event had happened to them and rate the stress thus experienced on a 10 point scale wherein 1 represented the least stress and 10 represented the most. Any other event, which had caused significant stress during the past one year, was to be mentioned in response to question 41. Several responses to question 41 were received including an inability to balance extracurricular activities and academics, loss of somebody else's borrowed belongings, commuting problems etc. However, none of them were included in the formulated scale, which was used for phase 2 as the items could be either included in one of the items already existing in the scale, were chronic / ongoing in nature or were too vague to be included.

One of the important feedbacks received at this stage was the reported difficulty in rating the stress on a 10 point scale. In the second phase of the study, a formulated 5 point scale similar to the one used in phase 1 was used. However, this time, the students were told to report only the events which had occurred in their lives in the past one year and then rate them. The sample size for this phase was 102 students, who did not overlap with the participants of phase 1 but were age-matched with phase 1 students. This data was used for validation of the scale using statistical measures. The validated scale was then administered to the third, simple, random sample of 123 students from the same schools, who also received CBCLs[1] to be filled by either parent after informed consent. Also, 100 out of these 123 students received a presumptive stressful life event scale[34] to be answered by the parents regarding stressful events which had occurred in their lives in the past one year. Parents of 8.9% of the adolescents refused to participate in the study. A database was created in MS Access and SPSS version 13 was used for analysis. Appropriate statistical measures and tests were carried out.

## RESULTS

The initial sample of 156 students consisted of adolescents-males and females ranging from 12—17 years of age [Table 1]. This was the phase wherein the students rated the imaginary events on a 10 point scale. A poor response rate was obtained for events like pregnancy, sex problems, marriage, jail term and excessive alcohol or drug use by a family member. Mean stress scores were calculated for each event depending on the results obtained [Table 2]. This gave an estimation of the stress perceived due to that particular event. The questions were statistically grouped via factor analysis into eight interpretable and mutually exclusive dimensions related to family and parent events, accident and illness events, sexual events, autonomy events, deviance events, relocation events, distress events and ambiguous events.

**Table 1:** Characteristics of the samples

	Mean age	No. of boys	No. of girls	Class
Formulation sample	13.673 yrs	81	75	8^th^, 9^th^, 11^th^
Validation sample	13.846 yrs	62	40	8^th^, 9^th^, 11^th^

**Table 2:** Life event stress scores

Events	Life change unit scores
New girl friend or boy friend	23
Outstanding personal achievement	39
Change in eating habits	26
Change in social activities	29
Change in sleeping habits	29
Change in living conditions	27
Outstanding achievement of sibling	31
Prophecy of astrologer or palmist etc.	21
Change in health of family member	40
Gain in a new family member	26
Mother stops or starts working	27
Change of major subject/ branch	31
Trouble with parents	29
Trouble with bullies	36
Death of pet	34
Parents unemployed	34
Increased workload at school	58
Change in financial status of parents	34
Theft of personal belongings	31
Dropped more than one class	33
Lower grades than expected	48
Serious argument with a teacher	37
Lack of attendance	29
Change of school	31
Sex problems	23
Marriage of an emotionally close sibling	24
Marriage	28
Excessive alcohol or drug use by family member	31
Jail term	32
Serious argument with a close friend	42
Minor violation of law	26
Appearing for an exam/ interview	46
Failed important course/ examination	42
Major personal injury or illness	35
Break up with a girlfriend/ boyfriend	32
Divorce between parents	43
Rustication from school	42
Death of a close friend	49
Death of a close family member	55
Pregnancy	23

Cronbach's alpha was calculated for each of the domains. Going by Fleiss's rule of thumb that a value of < 0.39 = poor, 0.40-0.74 = fair to good and 0.75-1.00 signifies an excellent validity, we found Cronbach's alpha for all our domains in excellent range except for the relocation domain for which it was in the fair to good range [Table 3]. The eight life events' domains were carefully examined by content in order to determine the amount of control exerted by the person over its occurrence. Items comprising of the family and parent events, relocation events, accident and illness events and ambiguous events were considered spontaneous and not under the choice or direction of individual. On the other hand, event clusters dealing with sexuality, autonomy and deviance included events that were considered to be under voluntary control of the subject.

**Table 3:** Cronbach's alpha scores

	Formulation sample	Validation sample	Events nos.
Sexual events	0.82	0.84	1,25,27,35,40
Deviance events	0.8	0.79	13,14,28,29,31,37
Relocation events	0.66	0.252	6,18,24^a^
Family/ parenting events	0.82	0.89	9,13,16,26,28,36
Ambiguous events	0.79	0.6	7,8,10,11,18
Distressful events	0.88	0.89	15,17,19,20,21,22,23,30,32,33
Autonomy events	0.76	0.42	12,3,4,5,2^b^
Accident/ illness events	0.84	0.7	34,38,39^c^

Factor/analysis

a. Variance e 6-52%, e18-32%, e24-16%;

b. Variance e12-36%, e5-23%, e4-18%, e3-12%, e2-11%;

c. Variance e34-63%, e38-27%, e39-10%

Though the distressful events scale contains items largely under the control of the individual (except for events like death of a pet or theft of personal belongings), the content is much more one of distress and response to pressures rather than isolated or independent events. Statistical verification of second order groupings of controllable and uncontrollable events was done and Cronbach's alpha for both the groups were found to be in an excellent range [Table 4]. Scores on uncontrollable events were found to be higher than controllable events though statistically not significant. (*P* = 0.355). Comparisons between ratings on the individual questions between the samples used for formulation (156 adolescents) and validation (102 students) using nonparametric tests, showed significant differences (*P* < 0.05) between the ratings on most of the events except for events 2, 9, 14, 18, 21, 22, 30, 32, 33, 36, 37, 38, 39. Ratings were higher on the sample reporting actual life events.

**Table 4:** Cronbach's alpha scores

Controllable events	0.84
Uncontrollable events	0.93

**Figure 1:**
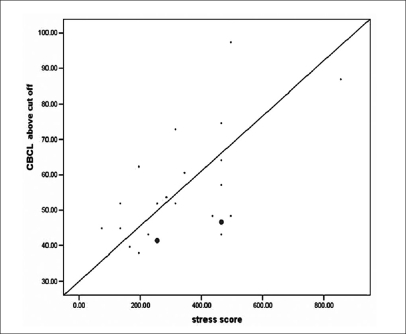
CBCL vs ALESS scores

There were no statistically significant differences between the scores on sample two and sample three on most of the events except events 9, 23, 30, 35. A positive correlation was found between the scores on the adolescent stressful life event scale and the presumptive stressful life event scale. This correlation was used to assess the stress caused in the parents in sample three due to life events although the strength of the correlation showed that it wasn't very high. (Pearson's coefficient = 0.011). As far as the CBCL scores were concerned, a strong positive correlation was found with the scores on the adolescent stressful life event scale. (Pearson's coefficient= 0.565, *P* = 0.004). Thirty-two percent variance of CBCL scores was found to be explained by the scores on the current scale. The formula which thus evolved for the calculation of CBCL scores from stress scores in the current study, is:

CBCL score (above cutoff) = 37.5 + 0.05 (stress score)

(as per regression analysis equation, y = a + bx [Figure 1])

## DISCUSSION

This paper addresses some psychometric properties of a new measure of potentially stress-causing life events in Indian adolescents. Development of a new instrument was driven by the need for an interviewer-based measure for the adolescents, especially meant for the Indian milieu that could be used in both community and clinical settings. Furthermore, it was hoped that such an instrument would permit the examination of antecedent events and stress caused due to them and their relationship to psychiatric disorders. With regard to the issue of a consistent and reliable measure of life events among adolescents, it was found that the 40 item-containing life event instrument revealed substantially similar internal qualities between two independent samples matched for age though varying in social class.

Although several scale variances and a few scale means differed, the corelational pattern among these factors was not statistically different. These findings indicate that the life event scale assessed similar qualities in both samples which gave us confidence in the substantive associations with other variables. The eight interpretable and mutually exclusive dimensions related to family and parent events, ambiguous events, accident and illness events, sexual events, autonomy events, deviance events, relocation events and distress events were found to be statistically valid. Thus, they encompassed the scale to delineate the sphere of life affected due to the life event. The area of life affected due to the event[17,18] has been considered to be an important facet of whether the event has been perceived and responded to as stressful.

The issue of control over the occurrence of life events has been addressed by several researchers[35,36] with mixed results. One drawback in much of the stress literature is the use of an overall summary score of life events rather than a taxonomy or clustering of events as some suggest.[17,18,35] Utilizing this approach of a multidimensional assessment of life events, we were able to group primary factors of life events into second order groupings related to uncontrollable stress events and controllable change events. This meaningful higher order clustering allowed us to test any differences in the stress experienced due to the two domains. Poor response rate to a few questions in the initial phase could be either due to the events being perceived as too far-fetched by the children or there was a deliberate attempt to conform to societal norms in not answering these “taboo” questions. Significant differences between the ratings on the individual questions between the samples used for formulation (156 adolescents) and validation (102 students) showed that the greater stress was associated with experienced events rather than with imaginary events.

These results are in contrast to previous findings that control subjects gave higher ratings on nonexperienced than on experienced events[37] or insignificant differences between the two.[34] This could be because of differences in the statistical measures used (nonparametric test for the current study vs t-test for the previous ones) or differences in population samples (Adult vs Adolescent population samples). Absence of statistical differences between samples 2 and 3 reinforce the belief in the internal validity of the scale. The internal consistency was measured and has been provided as Cronbach's alpha, which was found to be falling within the stardard of acceptable reliability. Tests of stability like inter- and intraobserver reliability were not required as the measure was self-administered.

The criterion of face validity represents a subjective judgement and thus even though ‘on the face of it, the instrument appeared to be assessing desired qualities’, it was not mentioned in the paper. As brought out in discussion, the instrument was found to sample all the relevant domains (content validity) but again was not mentioned on paper as face and content validity concepts are “validity by assumption”. The other aspects of validity and reliability will be assessed once this measure is used in other studies. The children identified by this study have now been followed up for more than a year. Now, the tests of diagnostic accuracy will be applied considering actual diagnosis as the gold standard.

When we consider adolescence as an age when the child is striving for independence, it is worthwhile to see the correlation between scores on adolescents' stress life event scale and those reported by the parents. Research has shown that negative life events reported by parents were associated with children's emotional and behavioral problems.[26,38] This correlation was determined to 1) assess the degree of overlap between the stress scores in the lives of parents and adolescents and 2) to assess if most of the stress experienced by adolescents could be accounted for by the significant life events happening in the lives of their parents. Though a positive correlation is found in the current study, not all the stress experienced could be accounted for as evident from a low positive value of the correlation coefficient. This, in turn, reemphasizes the role of adolescent-specific events in the lives of adolescents.

CBCL scores showing an increase with the scores on adolescent stressful life event scale, were a finding consistent with studies in the field of developmental psychopathology recognizing the potential importance of psychosocial stress in the etiology and maintenance of both internalizing and externalizing disorders in youth.[5–8]

### Limitations of the study

Scientific rigor adopted for the study resulted in high internal validity of the scale constructed. However, a limitation of this methodological study is that it was restricted to a sample of students attending to two schools. A selection of a simple random sample helped in overcoming this drawback and lent high internal validity to the scale. Life events are culturally loaded and thus, unless the measure is further validated, the findings based on this tool are also prone to various biases and threats.

### Confounding factors

Like in any other life event scale study, the retrospective recording of the events by the participants could have led to discrepancy in recording of the events. Also, high CBCL scores could be the cause rather than the result of stressful life events, especially the controllable events. However, no significant increase was found in controllable events in comparison to uncontrollable events in the current study in children with raised CBCL scores.

## CONCLUSION

In this study, we have attempted to look at several methodological problems in life events research in the context of understanding stressful events in adolescence. We have found that the current study's measure of life events appropriate for the teenage period was reliable and consistent across samples. A strong positive correlation of the current stress scale scores with CBCL scores, a model fit of r^2^ = 0.32 and an ability to predict the CBCL scores (above cutoff value) using the stress scores are some of the most important findings of this study.

We have been able to cluster events of life event scales into meaningful groups based on the life sphere affected and controllability of the events and detect mean stress scores for the events as they occurred in the lives of the adolescents in the past one year. Though the stress occurring in the life of the adolescent showed some degree of overlap with that of stress in the parent's life, a degree of independence was observed among the stress scores. With the current study we tried to devise a new scale to overcome the difficulties in administering foreign scales to our population of adolescents, which have resulted in a lot of irrelevant events being given importance and important events being left out.

We would again like to emphasize the emotional and financial dependence of an Indian adolescent on the family and the significance of family / parent-related events, which have been overlooked by many of the foreign scales. Evidence to date suggests that there is no simple relationship between adverse life events and the subsequent emergence of psychopathology. The interplay of acute and chronic stressors over the lifespan with affective temperament; the interrelationship of ‘sensitivity’ and ‘performance’ cognition in response to life events and limbic-cortical neural networks are all indicated as important avenues of future research.
